# Next-generation sequencing, phylogenetic signal and comparative mitogenomic analyses in Metacrangonyctidae (Amphipoda: Crustacea)

**DOI:** 10.1186/1471-2164-15-566

**Published:** 2014-07-06

**Authors:** Joan Pons, Maria M Bauzà-Ribot, Damià Jaume, Carlos Juan

**Affiliations:** Mediterranean Institute for Advanced Studies, IMEDEA (CSIC-UIB), c/Miquel Marquès 21, 07190 Esporles, Spain; Departament de Biologia, Universitat de les Illes Balears, 07122 Palma, Spain

**Keywords:** Crustacea, Amphipoda, *Metacrangonyx*, Mitogenome evolution, Mitochondrial RNA secondary structure

## Abstract

**Background:**

Comparative mitochondrial genomic analyses are rare among crustaceans below the family or genus level. The obliged subterranean crustacean amphipods of the family Metacrangonyctidae, found from the Hispaniola (Antilles) to the Middle East, including the Canary Islands and the peri-Mediterranean region, have an evolutionary history and peculiar biogeography that can respond to Tethyan vicariance. Indeed, recent phylogenetic analysis using all protein-coding mitochondrial sequences and one nuclear ribosomal gene have lent support to this hypothesis (Bauzà-Ribot et al. 2012).

**Results:**

We present the analyses of mitochondrial genome sequences of 21 metacrangonyctids in the genera *Metacrangonyx* and *Longipodacrangonyx*, covering the entire geographical range of the family. Most mitogenomes were attained by next-generation sequencing techniques using long-PCR fragments sequenced by Roche FLX/454 or GS Junior pyro-sequencing, obtaining a coverage depth per nucleotide of up to 281×. All mitogenomes were AT-rich and included the usual 37 genes of the metazoan mitochondrial genome, but showed a unique derived gene order not matched in any other amphipod mitogenome. We compare and discuss features such as strand bias, phylogenetic informativeness, non-synonymous/synonymous substitution rates and other mitogenomic characteristics, including ribosomal and transfer RNAs annotation and structure.

**Conclusions:**

Next-generation sequencing of pooled long-PCR amplicons can help to rapidly generate mitogenomic information of a high number of related species to be used in phylogenetic and genomic evolutionary studies. The mitogenomes of the Metacrangonyctidae have the usual characteristics of the metazoan mitogenomes (circular molecules of 15,000-16,000 bp, coding for 13 protein genes, 22 tRNAs and two ribosomal genes) and show a conserved gene order with several rearrangements with respect to the presumed Pancrustacean ground pattern. Strand nucleotide bias appears to be reversed with respect to the condition displayed in the majority of crustacean mitogenomes since metacrangonyctids show a GC-skew at the (+) and (-) strands; this feature has been reported also in the few mitogenomes of Isopoda (Peracarida) known thus far. The features of the rRNAs, tRNAs and sequence motifs of the control region of the Metacrangonyctidae are similar to those of the few crustaceans studied at present.

**Electronic supplementary material:**

The online version of this article (doi:10.1186/1471-2164-15-566) contains supplementary material, which is available to authorized users.

## Background

The metazoan mitochondrial genome (mitogenome) usually consists of a single compact circular DNA with a highly conserved gene content. It harbours the coding capacity for 13 proteins of four complexes of the respiratory chain, two ribosomal RNAs, and 22 genes coding for the tRNA set, including two gene copies for each leucine and serine tRNAs [1]. A non-coding region (control region) of variable length is also typically present (called d-loop in vertebrates and AT-rich non-coding region in arthropods); this region provides the site for the initiation of transcription and the initiation of replication of one or both strands [2–4].

A wealth of data on DNA sequence and gene organization of metazoan mitogenomes has been gathered in the last decades, with about 4000 complete mitochondrial genomes already deposited in DNA sequence databases (RefSeq release 64), of which two thirds correspond to vertebrates [5]. Data gathering has been fuelled by the advancements performed in “next generation sequencing” techniques and the need of generating robust phylogenetic information for evolutionary studies at all taxonomic levels, from deep metazoan evolution [6], relationships among vertebrate orders [7, 8], or even to resolve species-level phylogenies after rapid radiation processes [9, 10].

Up to now (May 2014) 134 crustacean mitogenomes have been completely sequenced, 107 of them corresponding to species in the class Malacostraca (NCBI RefSeq release 64 database). Within the malacostracan peracarid order Amphipoda, the sequences of species within the genera *Parhyale* (Hyalidae)*, Caprella* (Caprellidae)*, Onisimus* (Lysianassidae)*, Gondogeneia* (Pontogeneiidae), *Gammarus* (Gammaridae), *Eulimnogammarus* (Eulimnogammaridae)*, Pseudoniphargus* (Pseudoniphargidae), *Bahadzia* (Hadziidae) and *Metacrangonyx* (Metacrangonyctidae) have been reported or are deposited in sequence databases ([11] and references therein; NCBI RefSeq database).

In a previous work we sequenced the mitochondrial genomes of several Metacrangonyctid taxa by both classic and next-generation sequencing techniques to resolve, in combination with nuclear ribosomal sequences, the phylogenetic relationships within this family and to establish a time frame for its diversification [12]. The Metacrangonyctidae is a phylogenetically enigmatic amphipod lineage composed of stygobiont (occurring only in subterranean waters) species with an extreme disjunct geographic distribution [13]. Our phylogenetic reconstruction suggested that the major lineages of Metacrangonyctidae diversified during the Cretaceous, c. 96–83 million years ago (mya), and that the diversification of an insular clade was compatible with vicariance by plate tectonics [12]. In the present study we aim to analyse in more detail the mitochondrial DNA sequences of Metacrangonyctidae, their genome organization and evolution and compare them to other amphipodan mitogenomes. Aside of two species of *Caprella* [14, 15], the few known amphipod mitogenomes derive from species placed in distant genera and families. Here we analyse 21 metacrangonyctid mitochondrial complete or nearly complete genome sequences (including the 20 mitogenomes reported in [12] plus one previously obtained by us, *Metacrangonyx longipes* from Mallorca [16]), to explore their phylogenetic signal and to perform a comparative intra-familiar genomic analysis. We have compared them also with the mitogenomic features displayed by other amphipod families. We have paid particular attention to the role played by tRNAs and the secondary structure of the small/large ribosomal RNAs and their nucleotide substitution patterns, since few data are available for these genes in crustaceans.

## Results and discussion

### Genome organization

**Figure 1:**
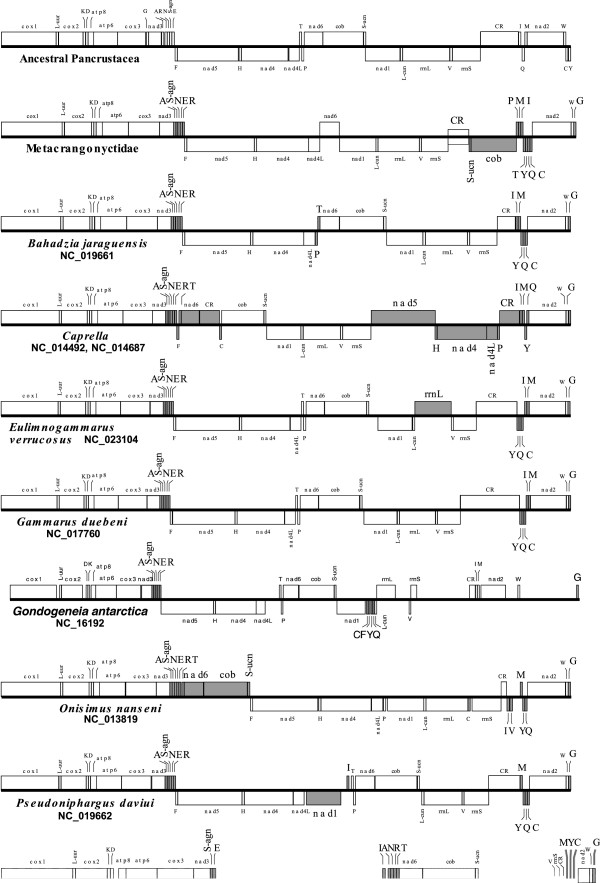
**Mitochondrial genome maps of Amphipoda.** Changes with respect to the hypothetical pancrustacean gene order are highlighted in grey. Genes located above the central line correspond to genes coded on the (+) strand, whereas those coded on the (-) strand appear below. The dashed segment of the mitogenome of *Parhyale hawaiensis* corresponds to accession numbers FM957525-6 [16]. The control region (CR) of Metacrangonyctidae is depicted in the middle line of the genome map to account for the uncertainty of in which strand lies the replication origin.

In previous studies we obtained the sequence information and complete or nearly complete mitochondrial genome of 21 metacrangonyctid specimens belonging to 10 species plus six taxa that are not formally described yet, covering the entire geographical range of the family (Table 1) [12, 16]. The sequence coverage of mitogenomes ranged from 4.3 to 5.7 × for the shearing/shotgun sequencing approach, to between 80–178 × and 59–281 × for the Roche GS Junior and Roche FLX approaches, respectively (see Table 1). All mitochondrial DNAs were circular molecules with a total size ranging (completed mitogenomes) from 14,113 (*M. longipes*) to 15,037 bp (*M. spinicaudatus*) (see Table 1). Genome content comprised the typical metazoan mitochondrial gene set consisting of 37 genes (13 protein-coding genes or PCGs, 22 tRNAs and two ribosomal genes), with 21 coded at the (+) strand and 16 at the (-) strand (Figure 1). Mitochondrial gene order in all taxa was identical to that previously reported for *M. longipes* [16], where *trnL2* (UUR) gene appears between *cox1* and *cox2* as in the putative pancrustacean (hexapods + crustaceans) pattern. This pancrustacean pattern is assumed to derive from a translocation of this gene with respect to the arthropod presumed ground pattern, but it shows also many rearrangements compared to the hypothetical ancestral pancrustacean gene order [16–18]. Gene arrangement in metacrangonyctids is unique among the Amphipoda although several gene blocks are conserved in most amphipods [16] (Figure 1). The mitogenomes of other amphipods known, such as those of *Parhyale hawaiiensis* [19, 16], *Caprella scaura* and *C. mutica* [14, 15], *Gondogeneia antarctica* [20], *Onisimus nanseni* [21], *Gammarus duebeni* [11], *Pseudoniphargus daviui* and *Bahadzia jaraguensis* [12], all show the gene string *cox1,trnL2* (UUR)*,cox2,trnK,trnD,atp8,atp6,cox3* conserved, except for a transposition of *trnK,trnD* in *Gondogeneia antarctica* (Figure 1)*.* In addition, the pancrustacean gene order *nad5,trnH,nad4,nad4L* is conserved at the same location of the (-) strand in all amphipod mitogenomes studied (and it appears in other non-pancrustacean invertebrates as well). However, in *Caprella scaura* and *C. mutica* this gene arrangement is placed in a different position between the *rrnS* gene and the control region, with the gene *nad5* on the (+) strand. All amphipod species but *P. hawaiiensis* display also the string *trnA*,*trnS1 (AGN)*,*trnN*,*trnE*,*trnR*, so this probably represents an apomorphic feature of the Amphipoda [11]. All metacrangonyctid mitogenomes have the particularity of having the *trnS2* (UCN) (not found in the original annotation of the mitogenome of *M. longipes* [16] but that has been annotated herein; see below) and *cob* genes coded at the (-) strand next to the control region, flanked at the other side by a string of tRNAs and the *nad2* gene. This gene order differs from the putative pancrustacean gene arrangement and is unique among amphipods (Figure 1). The breakpoint distances (dissimilarity function based on deduced gene rearrangements) calculated with CREx [22] between the hypothetical pancrustacean gene order and the amphipod gene arrangements are high, with a minimum distance of 13 (*Gammarus duebeni*) and a maximum of 21 (*Gondogeneia antarctica*) (results not shown). Therefore many rearrangements have occurred in this lineage making it difficult reconstructing the ancient events. The mitogenome of Metacrangonyctidae have suffered at least four tRNA gene transpositions (*trnN, trnR, trnC and trnG* genes), the inversion of *trnT,* one inversion coupled with transposition of the string *trnS* (UCN),*cob* and two complex tandem duplications with subsequent random gene loss. The more similar gene orders are the ones of *Gammarus duebeni* and *Eulimnogammarus verrucosus* (both within the superfamily Gammaroidea) only differing in the orientation of the *rrnL* gene (Figure 1)*.*

**Table 1:** **Metacrangonyctidae mitochondrial genome information**

Species	Code	Accession number	Sequencing method	Average read length	Coverage	Length	A + T	AT skew	GC skew
*M. longipes* (Mallorca)	LON	AM944817	Sanger	N/A	N/A	**14113**	76.029	-0.017	-0.035
*M. longipes* (Menorca)	SFM	HE861923	GS Junior	437	87×	**14117**	74.995	-0.015	-0.042
*M. repens*	REP	HE860495	Sanger	550	5.7×	**14354**	76.878	-0.025	-0.014
“L. stocki” (Tafraut)	HOM	HE860496	GS Junior	395	160×	12924	73.336	-0.099	0.174
“M. boutini boutini”	BBM	HE860497	GS Junior	433	107×	13301	69.799	-0.078	0.152
“M. boveei”	VOB	HE860498	GLX	511	100×	**15012**	72.589	-0.009	0.005
*M. dominicanus*	DOM	HE860499	Sanger	625	4.3×	14536	73.652	-0.016	-0.026
*M. goulmimensis* (Erfoud)	LMG	HE860500	GS Junior	413	178×	14503	69.737	-0.016	-0.028
*M. goulmimensis* (Ousroutou)	OUM	HE860501	GS Junior	454	100×	**14602**	74.873	-0.020	-0.037
*M. goulmimensis* (Zouala)	ZOM	HE860502	GS Junior	423	165×	**14352**	75.516	-0.028	-0.046
*M. ilvanus*	ILV	HE860503	GLX	540	78×	14770	74.563	-0.014	-0.012
“M. nicoleae tamri” (Tamri)	TAM	HE860504	GLX	595	59×	**14644**	75.130	-0.062	0.120
*M. samanensis*	PFM	HE860505	Sanger	551	5.7×	14057	74.141	-0.016	-0.034
*M. spinicaudatus*	SKM	HE860506	GLX	534	124×	**15037**	74.789	0.010	-0.139
“M. paurosexualis”	SKP	HE860507	GS Junior	424	94×	12542	75.546	-0.037	0.024
“L. stocki” (Tiznit)	ASL	HE860508	GS Junior	431	103×	12747	72.519	-0.096	0.161
*M. longicaudus*	LML	HE860509	GS Junior	403	135×	14710	75.819	-0.014	-0.051
*M. panousei*	AGM	HE860510	GS Junior	453	80×	14478	76.150	-0.012	-0.051
“M. nicoleae tamri” (Aksri)	AKM	HE860511	GS Junior	411	145×	13517	74.018	-0.049	0.111
*M. remyi*	REM	HE860512	GLX	454	281×	14787	70.785	-0.014	0.017
“M. notenboomi”	MAM	HE860513	GS Junior	420	168×	14277	74.385	-0.019	-0.043

Numbers in bold indicate complete mitogenomes; rest of mitogenomes are only partial since some regions (usually the AT-rich region or a short fragment between *rrnS* and *rrnL* genes) were not sequenced due to technical problems. Species not formally described yet are quoted with a tentative latinized binomen within inverted commas, and not in italics.

### Base composition and AT- and GC-skews

**Figure 2:**
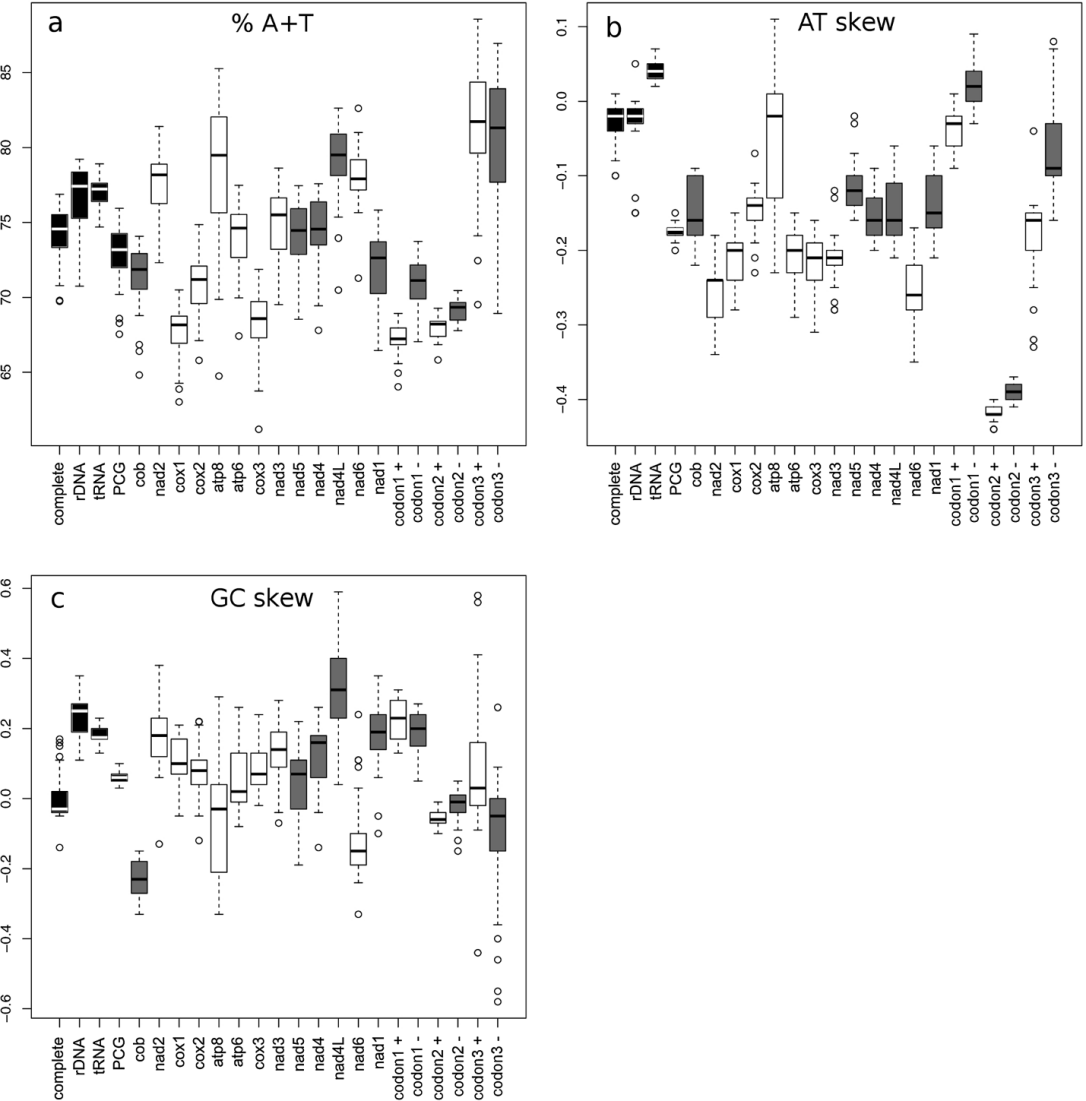
**Metacrangonyctidae mitochondrial nucleotide composition.** Box plots showing values of nucleotide composition (A + T percentage) **(a)**, AT-skew **(b)** and GC-skew **(c)** across mitogenomes (indicated as complete), for ribosomal (rRNA), transfer ribosomal (tRNA) and across protein coding genes (PCGs) as boxes filled in black. The same features are shown for each protein-coding gene and pooling by codon position and coding strand. Grey-filled boxes indicate genes coded at the (-) strand and empty boxes in the (+) strand.

The variation in AT% across metacrangonyctid taxa (species for which the entire genome was completed) ranges from 72.59 to 76.87%, with an average value of 75.09% (Table 1). This high AT richness is typical of hexapodan species and appears also in many crustacean mitochondrial genomes [23]. The average whole mitogenome AT-skew was -0.02, with a variation ranging from -0.06 to +0.01. The average GC-skew was -0.02 (-0.14 to +0.12) with most mtDNAs displaying negative skews (Table 1). Figure 2 graphically depicts the AT%, AT- and GC-skews for the entire mitochondrial genomes across species. Average AT% variation for the protein-coding genes in all metacrangonyctid species ranged from 67.54 (*M. goulmimensis* Erfoud) to 75.94 (*M. repens*) (Table 1, Figure 2a). Three species (*M. goulmimensis* Erfoud, “M. boutini boutini” and *M. remyi*) displayed similar significantly lower AT-content (of about 68%). No particular trend was observed in AT-content of genes placed at different strands. The *atp8* gene showed the largest variation in AT-content across species. Two species, *M. goulmimensis* Erfoud and “M. boutini boutini” displayed outlier lower AT% values for most of the protein coding genes.

Metazoan mitogenomes show a marked strand bias in nucleotide composition, which is thought to be due to exposure to different mutational pressures during replication, transcription or during both processes [24]. Most malacostracan mitogenomes exhibit a negative GC-skew for genes coded in the (+) strand and positive values for genes of the (-) strand [11]. The Isopoda (which, as the Amphipoda, belong to the superorder Peracarida) seem to be an exception to this rule since their mitogenomes show a reversed pattern where most genes of the (+) strand have a positive GC-skew; i.e. more G than C. Nevertheless, we have found that metacrangonyctid amphipod mitogenomes show GC-skew positive values at both (+) and (-) strands (Figure 2c), with the exception of *cob* and *nad6* genes. AT-skew values are in turn negative for all protein-coding genes but *atp8* (Figure 2b), with genes coded on the (+) strand showing lower overall values than those coded on the (-) strand. The reversed strand bias pattern of isopodan mitogenomes and in general any other metazoan strand bias have been explained advocating to the occurrence of an inversion of the control region. This inversion presumably included the replication origin at the base of Isopoda, changing the mutational pressure leading to strand-bias [24–26]. The control region is placed between the *rrnS* (- strand) and *trnY* (- strand) genes in the amphipod completed mitogenomes (with the exception of *Caprella,* that displays two A + T-rich regions at non-conserved positions, and metacrangonyctids). The segment assigned as the control region in metacrangonyctids is flanked also by *rrnS* (as in all amphipods except in *Caprella)* but *trnS2* (UCN), followed by the *cob* gene are at the other side. It can be deduced that both *trnS2* (UCN) and *cob* have suffered a reverse transposition (i.e. transposition plus strand switch) respect to the hypothetical arrangement displayed by other amphipods suggesting that this could have caused an inversion of the control region in the Metacrangonyctidae lineage. In the isopods *Eophreatoicus* sp. and *Ligia oceanica*, that show a similar strand bias pattern as metacrangonyctids, the control region appears between the *trnQ* and *trnI* genes (both coded at the (+) strand in *L. oceanica* while *trnI* is at the (-) strand in *Eophreatoicus* sp.). In any case, the strand bias pattern of metacrangonyctid mitogenomes is not only more similar to the condition found in the Isopoda than to amphipods, but also to other non-peracarid crustaceans such as *Hutchinsoniella macracantha* (Cephalocarida); *Tigriopus californicus, T. japonicus, Lepeophtheirus salmonis, Calanus hyperboreous* (Copepoda); *Argulus americanus* (Branchiura) and some decapods (*Procambarus claarkii, P. fallax, Corallianassa couitierei, Nihonotrypaea japonica, N. thermophila, Cambaroides similis, Homarus gammarus)*. All these species share with metacrangonyctids the display of a positive GC-skew in genes coded in the (+) strand (Additional file 1). This suggests that the reversal of the ordinary strand bias has occurred independently multiple times, and not only in very distant metazoans [24] but also within the Crustacea, even within members of the same taxonomic order. This is presumably due to the fixation of different independent ancestral inversions of the same block of mitochondrial genes with respect to the control region, or vice versa [24].Nucleotide composition per codon site showed a sharp contrast between third and first/second positions, as expected (Figure 2a). Third codon positions displayed a high AT-content with similar values at both strands (AT = 81.30% on (+) strand; 80% on (-) strand), and a large variation across species. In contrast, AT-content at the first and second codon positions were lower and differed in genes coded on different strands, in particular the first codon positions. AT skew was close to zero at first codon positions, second codon positions showed a T nucleotide-enrichment (about -0.4 AT skew value on average) in genes of both strands, whereas third codon positions showed intermediate negative AT skews. GC skew per codon position was positive for first codon positions, slightly negative or close to zero for second codon positions, showing a substantial variation for third codon positions (Figure 2c).

### Amino acid frequencies and codon usage

**Figure 3:**
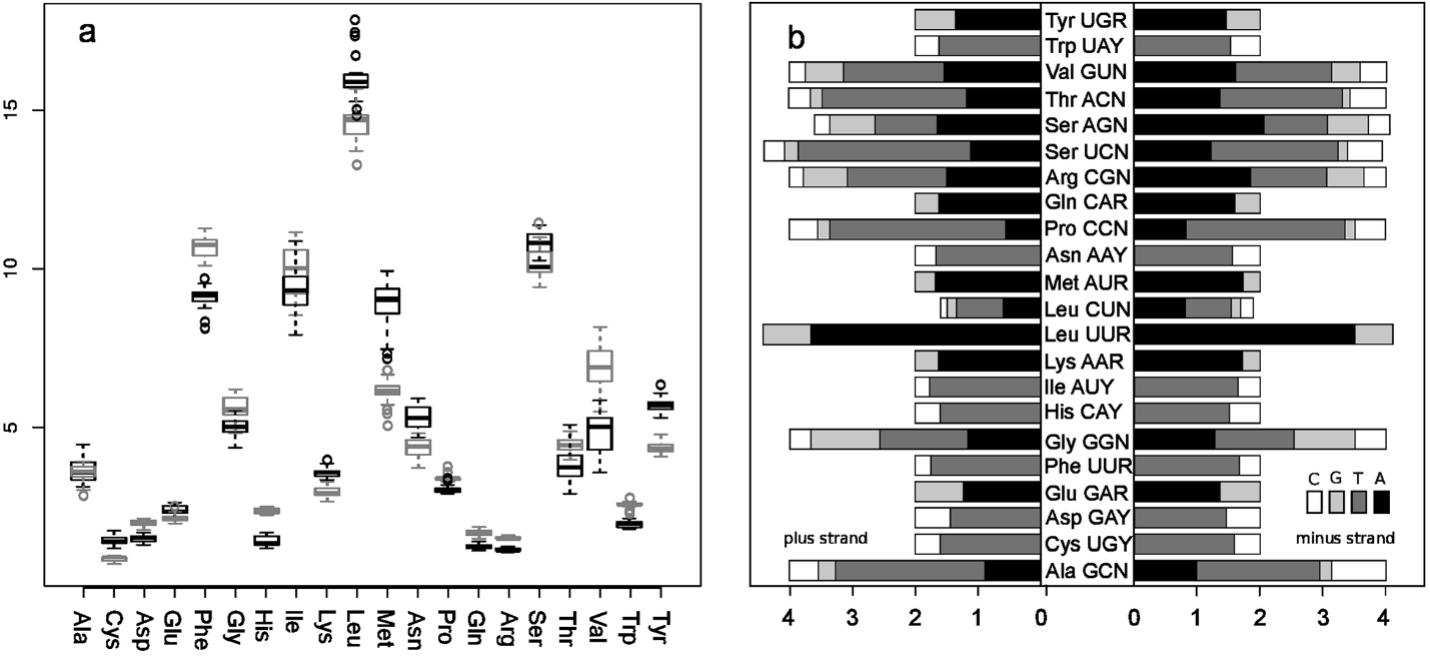
**Metacrangonyctidae mitochondrial protein-coding gene amino acid composition. (a)** Box plot showing amino acid composition for PCGs across mitogenomes. Grey and black boxes display values estimated for genes coded on the (+) and (-) strands, respectively. **(b)** Relative Synonymous Codon Usage (RSCU) for genes coded on (+) and (-) strands.

The analysis of amino acid frequencies indicate that five amino acids account for more than half of the total amino acid composition (leucine, phenylalanine, isoleucine, serine and methionine). Overall amino acid frequency patterns were similar irrespective of the coding strand, with the more used amino acids showing a higher variation across species (Figure 3a). Similarly, a measure of the extent that synonymous codons depart from random usage (computed as relative synonymous codon usage values) showed a conserved pattern in both strands, evidencing the high prevalence of A or T nucleotides at third codon positions (Figure 3b). The Effective Number of Codons (ENC) is another measure of the synonymous codon usage bias [27]. ENC values averaged 40 ± 3.33 for the (+) strand and 42.25 ± 3.65 for the (-), indicating that only about two-thirds of the possible codons are used in metacrangonyctid mitogenomes. The positive correlation between ENC and GC content at third codon positions has been reported also in other mitochondrial genomes (Additional file 2) [25]. The influence of a strong compositional bias for A + T and highly biased codon usage has been also recently described in aphid mitogenomes [28].

### Protein coding gene phylogenetic informativeness

**Figure 4:**
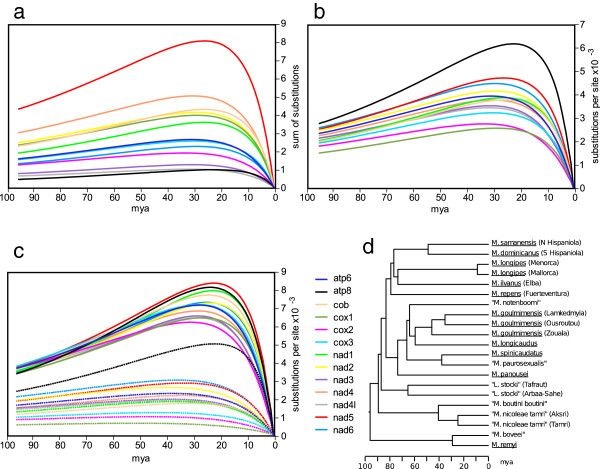
**Metacrangonyctidae mitochondrial protein-coding gene phylogenetic informativeness.** Chronological measure of Phylogenetic Informativeness (PI) for each protein-coding gene as a net **(a)** and per site **(b)** rate values. Panel **c** shows PI values per site for third codon positions (solid lines) and for first and second codon positions combined (dotted lines). **(d)** Metacrangonyctid bayesian phylogenetic chronogram based on nucleotide protein-coding gene sequences reported in [12].

The Phylogenetic Informativeness approach (PI) aims to determine the power of a gene (or a set of characters) to resolve branching order in a particular time frame in a phylogenetic tree [29]. PI values for the 13 PCGs are relatively high from phylogenetically recent time up to 20–25 mya in the metacrangonyctid phylogenetic tree obtained elsewhere [12], with third positions providing most of the phylogenetic informativeness (68%) (Figure 4c). Saturation of nucleotide substitutions is mostly concentrated at third codon sites as expected, with phylogenetic signal dropping deeply in the phylogenetic tree from 25 to 96 mya. First and second codon sites, however, appear to maintain most of their weak phylogenetic signal through time. Comparison of the PI values for the different protein-coding genes confirms the expectation that there is a positive correlation between phylogenetic informativeness and gene length. However, if a correction by length is applied, the *atp8* gene shows the highest PI value, followed by *nad5, nad6* and *nad2* (Figure 4b). On the other hand, the less informative genes in terms of phylogenetic content when we take into account gene length are *cox1, cox2* and *cox3*. Interestingly, *atp8* gene shows the highest PI for the first and second codon positions combined compared to any other PCGs (Figure 4c). We can conclude that the genes with more accumulative phylogenetic informativeness are *nad5, nad2, nad1, cob* and *nad6*, while *cox1* and *cox2* are relatively poor in phylogenetic informativeness despite their prevalence in phylogenetic reconstruction studies. Havird and Santos [30] have recently analysed a large metazoan mitogenomic data set concluding that *nad5, nad4* and *nad2* genes were the more likely to reproduce the phylogeny obtained from concatenation of all 13 PCGs, while the popular marker *cox1* and some of the other long PCGs were the less phylogenetically reliable at this deep taxonomic level. Although particular genes can provide phylogenetic power at distinct divergence time intervals and be more informative in some lineages than others, our data agree with the analysis by [30] in that genes of the NADH dehydrogenase subunits have in general more phylogenetic power than genes coding for the cytochrome subunits.

### Non-synonymous/synonymous substitution rates

**Figure 5:**
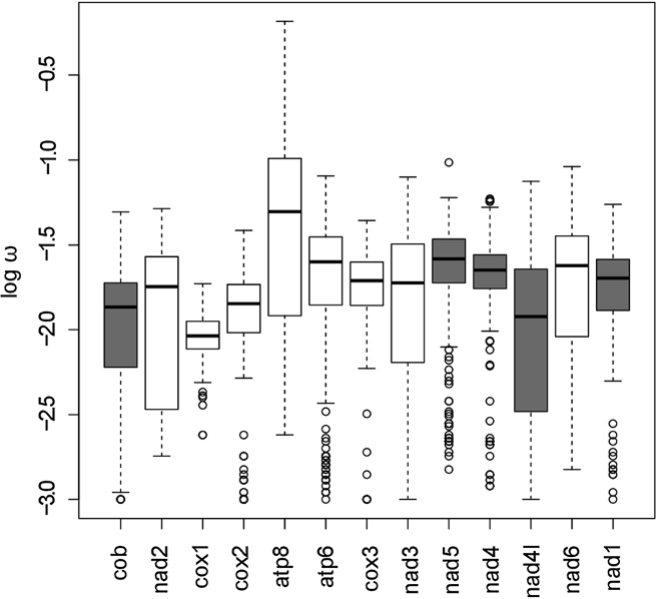
**Nonsynonymous/synonymous substitution ratio.** Box plot for the nonsynonymous/synonymous ratios (log ω = dN/dS) for the mitochondrial protein-coding genes of Metacrangonyctidae. Empty and filled boxes display values estimated for genes coded on the (+) and (-) strands, respectively.

The PCGs of the 21 mitogenomes were used to estimate dN/dS ratios (the ω parameter) by maximum likelihood, assuming rate constancy per codon site and branch. The ω value is a proxy of the intensity and type of natural selection acting on a particular protein, with expected values of ω as < 1, = 1, or > 1 under negative (purifying) selection, neutral evolution, or positive (darwinian) selection, respectively. Figure 5 shows the pairwise ω estimates for each of the 13 genes of the metacrangonyctid mitochondrial genome using the species phylogeny obtained by [12]. All ω values fall well below 1, suggesting that metacrangonyctid mitochondrial protein-coding genes are under purifying selection, with *cox1* and *atp8* genes being submitted to the strongest and weakest negative selection, respectively. This has also been shown to be the case in other mitochondrial comparisons such as those established among parasitic *Nasonia* wasps [31], aphids [28], *Xenopus* species [32] or among other vertebrate mitogenomes [33]. Oliveira et al. [31] noticed that *atp8* amino acid substitutions accumulate three times faster than average mitochondrial PCGs in *Nasonia,* suggesting that either positive or relaxed selection is acting on this gene. A similar pattern is observed for vertebrate mitogenomes, where the genes of the respiratory complex V (*atp6* and *atp8*) are under the least efficient selection while cytochrome b (*cob*) and cytochrome oxidase genes (*cox*) of the III and IV respiratory complex are submitted to the most efficient purifying selection [33]. In Metacrangonyctidae mitogenomes *atp8* ω pairwise comparisons vary extensively (ω values ranging from 0.002 to 0.656), showing an overall 0.074 value across the phylogenetic tree. This value is almost seven times higher than the one estimated for *cox1*, the slowest evolving gene in the metacrangonyctid lineage in terms of non-synonymous substitutions, with ω = 0.011 across the tree. This pattern is in accordance with the phylogenetic informativeness of both genes.

### Start and stop codons

Most PCGs displayed ATN start codons, with ATG and ATT as the most frequent (Additional file 3: Table S1). The rest of start codons found are considered as canonical for invertebrate mitochondrial PCGs, such as TTG, which is conserved in all metacrangonyctid mitogenomes except in *M. remyi* that shows the non-canonical CTG. In addition, the canonical start codon GTG is present in the *atp8* gene of two species (*M. remyi* and *M. repens*) instead of the ATN displayed in the rest of metacrangonyctids. The TAA complete or incomplete TAa or Taa stop codons are usually the norm in metacrangonyctid mitogenomes, although TAG appears as the stop codon for the gene *nad3* in the majority of species (Additional file 3: Table S1). Incomplete stop codons are believed to be completed by post-transcriptional polyadenylation [34].

### tRNA structures

**Figure 6:**
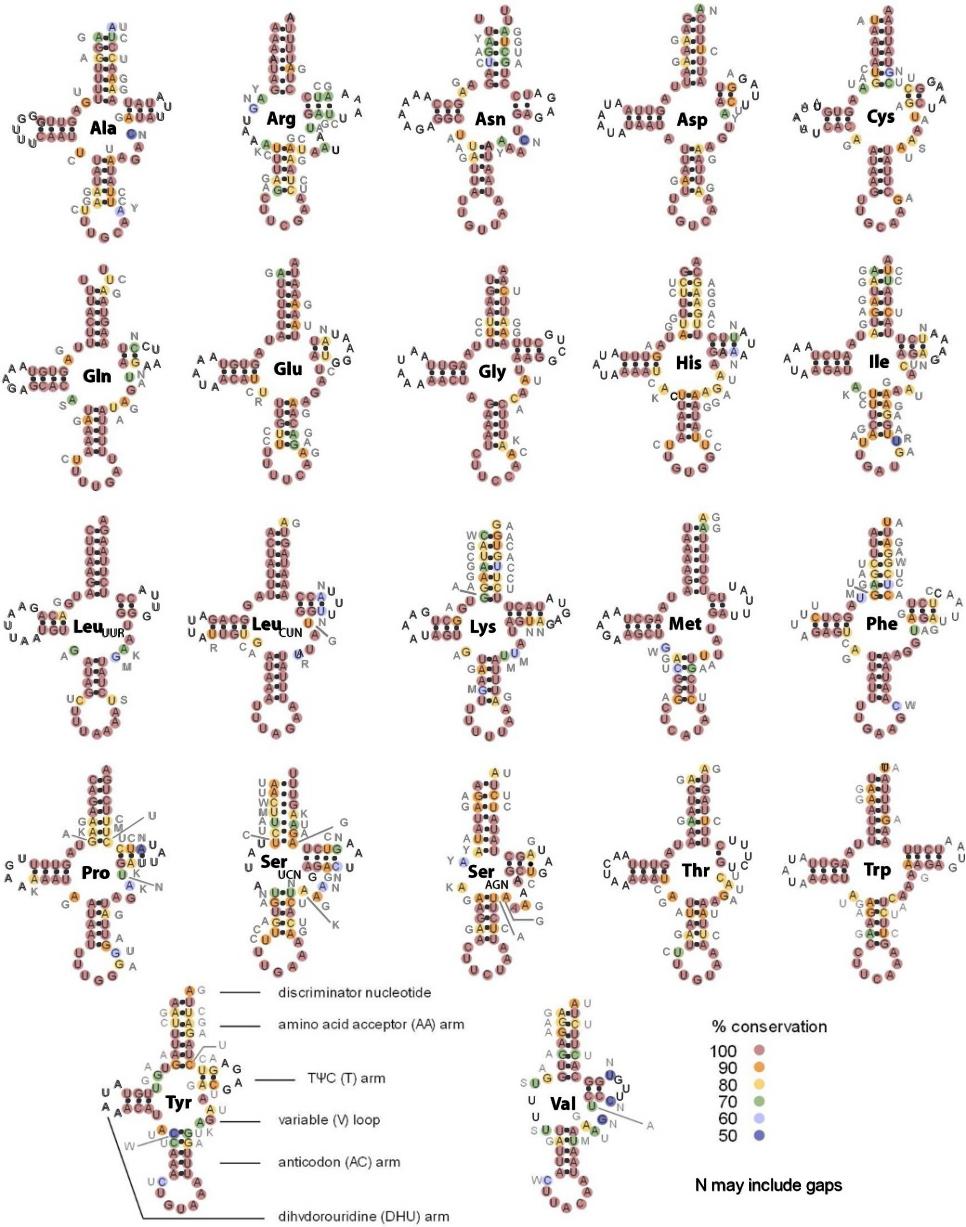
**Mitochondrial tRNAs structures.** Secondary structures predicted for the 22 tRNAs of “Metacrangonyx boveii”. Nucleotide conservation across metacrangonyctid mitogenomes based on multiple alignments is depicted with different colours. Nucleotide changes are indicated with letters in grey. Changes on DHU and TΨC loops are not indicated since many indels are concentrated at these segments.

We identified all the expected 22 tRNA genes in the metacrangonyctid mitogenomes, some of them only after implementing a -30 COVE score cut off and using bacterial or nematode mitochondrial tRNA models that lack the DHU or the TψC-loop arms. In this analysis, we annotated the gene *trnS1* (AGN) of *M. longipes* Mallorca that was not found in the previous search [16]. All sequences could be folded into typical cloverleaf structures showing the anticodon triplets, although several lacked the DHU arm (see below and Figure 6). Thirteen tRNA genes were on the (+) strand while nine were on the (-) strand (Figure 1). tRNA length ranged from 50 to 64 bp in the reference species “Metacrangonyx boveei”. Figure 6 shows the secondary structures of the reference species and the conservation of primary sequence among metacrangonyctid tRNAs. Four tRNAs (tRNA-Arg, tRNA-Ser_UCN_, tRNA-Ser_AGN_ and tRNA-Val) lacked the DHU stem, a helix that is highly conserved in the primary structure of other tRNAs. Aberrant secondary tRNA structures with missing DHU and TΨC stems have been reported in other amphipods [11]. The DHU-domain of tRNA-Ser_UCN_ has been lost in almost all metazoans, while for tRNA-Ser_AGN_ has occurred preferentially within particular Lophotrochozoan and Ecdysozoan lineages, probably due to independent loss events [35]. The basic conserved structure of the remaining 18 tRNAs is composed of the amino acid acceptor stem (7 bp), the DHU stem (3–4 bp), DHU loop (3–7 nts); anticodon stem (7 bp) and loop (7 nts); variable loop (4–5 nts) and TΨC stem (1–4 bp) and loop (3–5 nts). The amino acid acceptor and DHU stems appear always separated by two nucleotides and the latter from the anticodon stem by one nucleotide. The two genes specifying tRNA-Ser were relatively variable in sequence (20 and 26 nucleotide substitutions observed for tRNA-Ser_UCN_ and tRNA-Ser_AGN_, respectively) showing only a high conservation of the anticodon loop sequence, while the two genes for tRNA-Leu were the most conserved (showed only 8–9 substitutions) (Figure 6). No clear conservation pattern was observed with respect to placement of genes on the (+) or (-) strand. Based on the secondary structure estimation, 43% of nucleotide substitutions were deduced to be A < -- > G changes, 28% were T < -- > C and 19% A < -- > T, with most substitutions deduced to correspond to compensatory mutations occurring at stems (i.e. an A-U pairing in one species or group of species substituted by a G-C pairing in others). Compensatory nucleotide substitutions have been previously described in other ribosomal sequences such rRNAs and viral RNA [36, 37]. Several mismatched pairs were observed at stems (e.g. U-U in the amino acid acceptor arm of tRNA-Asn, which is conserved across the 21 studied tRNAs; and U-U or U-C in the amino acid acceptor arm of tRNA-Gln). It has been proposed that post-transcriptional mechanisms can mend and transform into fully functional those tRNAs with non-Watson-Crick matches or displaying other aberrant characteristics [38].

### Mitogenomic spacers

A total of eighteen different sequence spacers (isA-isR) were inferred to occur across the studied mitogenomes. Their length varied between 1 and 246 bp, although most of them were short 1–3 bp spacers (Additional file 3: Table S2). Six genome spacers appear to be conserved since they are placed in the same position in almost all species, suggesting an ancestral common origin although the primary sequences are not conserved. Two of these spacers are intergenic sequences separating PCGs: isH has 1 or 3 bp and is situated between *cox3* and *nad3* and isL, placed between *nad6* and *nad1*, varies from 2 to 246 bp. The mitogenome of *M. goulmimensis* (Zouala) has the longest isL due to 22 perfect repeats of the motive AAATTTATTT flanked by non-repetitive regions of 14 and 12 bp, respectively, while the spacer in the mitogenome of *M. goulmimensis* (Erfoud) forms a palindrome capable of forming a stem of 26 bp. In turn, the isL spacer of “M. notenboomi” is 73 bp long, of which 51 bp possibly derive from a duplication of the 3′ end of the gene *nad1* (88% similarity). All other genomic spacers are located between tRNA genes. The mitogenome of *M. spinicaudatus* shows a unique internal spacer (isN) of 173 bp between *trnR* and *trnF* genes; it could have originated from duplication since it shows a 73% similarity with gene *trnD*. The long inverted repeats present in mitogenomic spacers have been interpreted in some cases as an extra origin of replication ([28] and references therein). In other cases, spacers could be just remnants of a duplication process produced by slipped strand mispairing or imprecise termination during replication [39].

### Control region

The non-coding unassigned region located between the *rrnS* and *trnS2* (UCN) genes in all mitogenomes show the expected characteristics of control regions such as a high A-T content, presence of a secondary structure with T-rich loops, plus repetitive elements and palindromes [40]. This region displays also the lowest GC-skew, a feature indicative of the presence of the origin of replication [41]. The complete control region was obtained for eight mitogenomes and showed an AT-richness in the range 85.5-100% and lengths between 25 and 963 bp. Although variable in size and sequence, all control regions showed five common features [41]; namely: i) a TATA motif followed by ii) a 14–15 poly-T stretch; iii) a variable region (absent in some mitogenomes) capable of forming one or several stem-loop structures; iv) a 10–12 poly-A stretch and v) a GANT motif embedded in the *trnS*_UCN_ gene (Additional file 4). The variable region of *M. goulmimensis* (Ousroutou) comprises one motif of 216 bp followed by the sequence in inverted orientation (97% sequence identity lacking indels, Additional file 4). *M. spinicaudatus* has flanking repeats with inverted orientation of 93 bp (84% sequence identity without indels, not shown). Seemingly, “M. boveei” shows a long inverted repeat of 378 bp (100% identity and 11 indels, Additional file 4). In some mitogenomes these palindromes could have in part originated from a short tandem repeat, such as in the mitochondrial genome of *M. goulmimensis* (Ousroutou) where five monomers of 24 bp show a 74.2% identity (motif T_7_A_7_TA_9_). Two of the mitogenomes (*M. longipes* Menorca and *M. goulmimensis* Zouala) showed minimal and similar control regions with the motif (T)_14–15_(A)_13–15_ as they lack the variable region and show a secondary structure identical to the one deduced for *M. longipes* (Mallorca) [16] (Additional file 4). Similar structures have been described in the control regions of the isopods *Armadillium vulgare* and *A. pelagicum* [41].

### *RrnS*and *rrnL*structure

**Figure 7:**
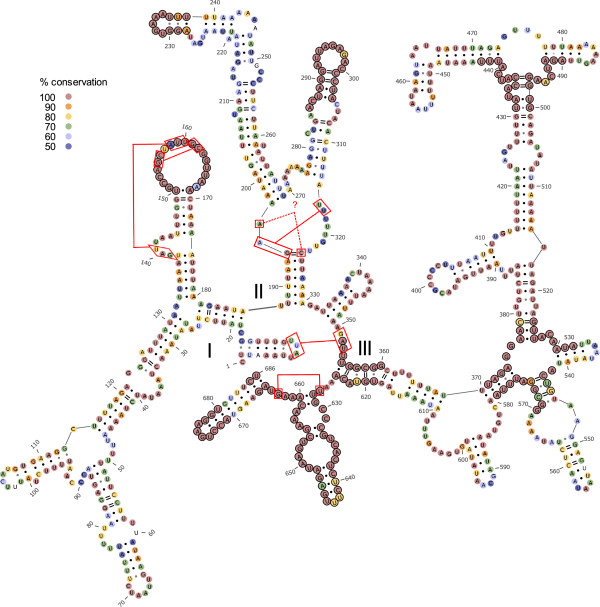
**Mitochondrial small ribosomal RNA structure.** Predicted secondary structure of the mitochondrial small ribosomal unit (12S rRNA) of “Metacrangonyx boveii” based on that estimated for *Artemia franciscana*. Nucleotide conservation across metacrangonyctid species derived from multiple sequence alignments performed with PRALINE is depicted with different colours. Positions conserved relative to *Artemia franciscana* are denoted with a circle. Red lines and boxes show positions deduced to be involved in tertiary folding. Different domains are labelled with roman numerals.

**Figure 8:**
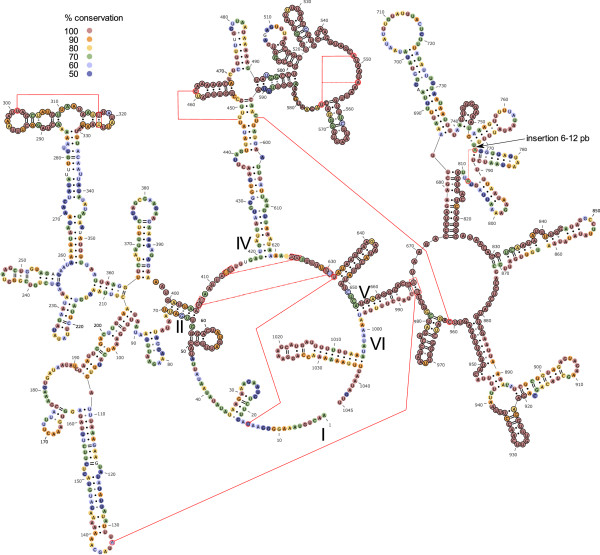
**Mitochondrial large ribosomal RNA structure.** Predicted secondary structure of the mitochondrial large ribosomal unit (16S rRNA) of “Metacrangonyx boveii” based on that estimated for *Artemia salina*. Nucleotide conservation, tertiary folding nucleotide interactions and domains as in Figure 7.

Metacrangonyctid mitogenomes show the large and small rRNA subunits placed as in the ancestral pancrustacean gene order [28], i.e. between the *trnL*1 (CUN) and the *trnV* genes (large subunit) and between the *trnV* and the control region (small subunit)*.* The boundaries of the two rRNAs were tentatively established in our taxa by alignment with published annotated ribosomal RNAs, although the 5′ and 3′ ends were found to be quite divergent among taxa. The *rrnS* sequences ranged from 624 to 693 nts, while the gene *rrnL* attained lengths between 1045 and 1068 nts. The predicted structure of the small and large mitochondrial RNAs of the species “Metacrangonyx boveei”, taken as representative of the Metacrangonyctidae, are shown in Figures 7 and 8, respectively (a dot-bracket notation is supplied in Additional file 3: Table S3). These, to our knowledge, are the first predicted mitochondrial ribosomal structures ever shown for an amphipod, and the second for a crustacean. The deduced *rrnS* structure shows the three conserved domains displayed by metazoan 12S RNAs and in particular by the crustacean *Artemia franciscana* (http://www.rna.ccbb.utexas.edu/RNA/Structures/b.16.m.A.franciscana.bpseq) [42] and all insect species whose 12S RNA structure has been determined [43] and references therein) (Figure 7). Despite the considerable sequence dissimilarity between both taxa, there are several highly conserved sequence motifs common to *Artemia* and the metacrangonyctid 12S RNA; namely: in the loop at domain I presumably involved in tertiary folding (positions 150–169), in three helices at domain II and in most of the primary sequence of domain III (see Figure 7). The high conservation of primary sequences and structure in domain III has been reported also from hexapod mitogenomes [28, 44]. The fourteen complete metacrangonyctid *rrnS* sequences obtained showed an average 76.9% AT content. The multiple alignment consisted of 721 positions, of which 358 were conserved (46.9%), with domain III being the most conserved region (61.1%). Figure 7 depicts the pattern of conservation of particular positions among metacrangonyctid *rrnS* sequences, suggesting that regions conserved in *Artemia franciscana* are also highly conserved in the different metacrangonyctid species. Notice that the nucleotide positions capable of interacting into tertiary structures are coincident with those proposed for the *Artemia franciscana rrnS* structure*.*

*The rrnL* sequences were similarly AT-rich (76.5% on average) and the inferred secondary structures showed the five canonical domains (I-II, IV-VI) displayed in all metazoans and absence of domain III as in all arthropods [43] (Figure 8). The metacrangonyctid *rrnL* multiple alignment comprised a total of 1105 positions, of which 475 were conserved (43.0%). Domains I and II were the most variable, showing only 30 and 25% identical positions, respectively, while domains IV, V and VI were more conserved (52-54% conserved positions) (Figure 8). A similar conservation pattern is shown in neuropterid insects 16S RNAs [43].

## Conclusions

The analysed metacrangonyctid mitochondrial genomes have a conserved gene order with a diagnostic translocation of the *trnS2* (UCN) and *cob* genes. This gene order differs from the pancrustacean gene arrangement and is unique among amphipods. In addition, PCGs show a reversed strand mutational bias pattern with GC-skew positive values at both strands except for two genes (*cob* and *nad6*), while codon usage seems to be influenced by base composition and strand mutational bias. The *atp8* gene displays the highest non-synonymous/synonymous rate ratio, being the more phylogenetically informative per position due to the frequent occurrence of non-synonymous changes at first and second codon positions. Purifying selection appears to have been stronger on genes of the cytochrome oxidase respiratory complex, in particular the *cox1* gene as shown for other mitogenomes. tRNA genes show a mutation dynamics similar to other metazoans, with frequent compensatory mutations at stems. Aberrant secondary structures lacking the D-stem have been determined in several metacrangonyctid tRNAs. AT-rich control regions, albeit quite variable in length, show common features and sequence motifs that can be related to their possible role as the replication origin. The *rrnS* and *rrnL* secondary structures of a reference *Metacrangonyx* mitogenome have been modelled based on the structures determined elsewhere in *Artemia* and sequence conservation within Metacrangonyctidae mapped on the obtained structures. These structures are similar to those shown in other arthropods, where conservation is concentrated at certain segment domains. To our knowledge these are the first *rrnS* and *rrnL* secondary structures determined for a peracarid and second for a crustacean.

## Methods

### Taxon sampling

We sampled specimens assigned to species of *Metacrangonyx* and *Longipodacrangonyx* from freshwater wells and caves spanning almost the entire known geographic range of the family. Material used in analyses was collected under collection permits issued to authors by the corresponding local or governmental authorities (i.e. Conselleria d’Agricultura, Medi Ambient i Territori, Govern de les Illes Balears; Consejería de Medio Ambiente, Gobierno de Canarias; Dirección de Biodiversidad y Vida Silvestre, Secretaría de Estado de Medio Ambiente y Recursos Naturales, Dominican Republic). No specific permits were required for specimens collected in Morocco and Elba Island. Several Moroccan taxa included in our analyses are not formally described yet are quoted with a tentative Latinized binomen within inverted commas and not in italics to identify this feature. Sampling locations with their corresponding geographic coordinates are listed in Additional file 3: Table S4. Major phylogenetic lineages within the family were identified based on partial mitochondrial cytochrome oxidase 1 (*cox1*) DNA sequences [12].

### Mitogenome amplification and sequencing

Long-range polymerase chain reaction (PCR) primers were designed on partial *cox1* and *rrnL* sequences to amplify the entire mitogenomes as described in [12] (primer list in Additional file 3: Table S5). Alternatively, the mitogenomes of four species were amplified as a single long fragment with primers targeting the *rrnL* and *rrnS* genes. Mitochondrial genomes were amplified from 50 ng genomic DNA using Herculase™ II Fusion DNA polymerase (Agilent, Santa Clara, CA, USA) following manufacturer’s recommendations, except for DNA fragments comprising the AT-rich region that only amplified using an extension temperature of 60°C. The mitochondrial sequence of three species was obtained using standard protocols [12, 45]. The mitochondrial sequences of the remaining species were obtained by next generation sequencing using Roche FLX/454 or GS Junior technology. In a first approach, the long PCR amplicons were purified using Invitek columns (Invitek GMBH, Berlin, Germany), quantified on a Nanodrop spectrophotometer and 5 μg (300 ng/μL) used for FLX tagged library preparation. The individual libraries of seven species were pooled in equimolar ratios and analysed in parallel in a pyrosequencing reaction using the Roche FLX/454 giving a total number of 200,000 reads corresponding to the output of 1/8th lane of the Roche FLX/454 sequencer. Methods for tagging and library preparation were previously described in [46] and followed manufacturer’s instructions. The sequence data obtained was sorted according to their tag sequences and preliminary assembled into seven subsets using the Newbler assembler. For twelve additional species we explored the simpler and cost effective multiplex sequencing method without the need of individual tagging by barcodes [47]. Firstly, we tested whether we correctly recover the previous mitochondrial sequences assembling from scratch in a pool of the total reads of the tagged library but after elimination of the species-specific sequence barcodes. This analysis demonstrated that mitochondrial sequence divergences were sufficiently high to reconstruct the whole mitogenome of each species without formation of chimerae. For these additional species, two batches of PCR amplicons representing six mitogenomes each were purified, quantified and pooled in equimolar ratios as above at a final concentration of 100 ng/ul per mitogenome. Each batch was sequenced as a single library in a Roche GS Junior giving a total number of about 100,000 reads per run. Sequences of *cox1*, *cob* and *rrnL* amplified from each species with universal primers and sequenced by standard Sanger method were included in the bioinformatic assembly of reads to confirm the correctness of assignment of each mitochondrial sequence (“bait” sequences as in [47]). Final assemblies from the FLX and GLS platforms were based on minimum sequence coverage of 59 ×.

### Mitogenome assembly, annotation and analyses

DNA sequence read quality filtering, contig assembly and gene annotation were performed as described in [12] with tRNA structures refined with tRNAscan-SE v1.21 (http://lowelab.ucsc.edu/tRNAscan-SE/) and checked using the MITOS webserver (http://mitos.bioinf.uni-leipzig.de/help.py) [48]. The annotation of start and stop codons plus tRNAs secondary structures were accomplished after exhaustive comparisons among the obtained mitogenome sequences. Gene rearrangements with respect to other amphipod mitogenomes or to the hypothesized pancrustacean ancestral gene order were deduced using strong interval trees on the CREx webserver (http://pacosy.informatik.uni-leipzig.de/crex) [22]. Nucleotide and amino acid composition plus codon usage profiles (Relative Synonymous Codon Usage RSCU) were estimated with MEGA v5.10 [49]. AT and GC skew were estimated as follows: ATskew = (A-T)/(A + T) and GCskew = (G-C)/(G + C) [50]. Effective number of codons (ENC) were determined taking into account background nucleotide composition as implemented in INCA v1.20 [51]. DNA direct and inverted repeats in spacer regions were explored with the EMBOSS package v6.5 (http://emboss.sourceforge.net/) [52] with the tools *einverted*, *palindrome* and *etandem*. The phylogenetic informativeness (PI) of protein-coding genes was estimated using PhyDesign (http://phydesign.townsend.yale.edu/) [29]. This method estimates maximum likelihood values per site on a tree topology derived from protein-coding sequences with each codon position considered as an independent partition [29]. The tree topology obtained in [12] was used for the PhyDesign analysis. Non-synonymous/synonymous substitution rate analysis (dN/dS) of the protein-coding genes was performed with the basic codon substitution model [53] in the PAML v.4.7 software package [54]. Nucleotide frequencies were calculated in the analysis from the average nucleotide frequencies at the three-codon positions (CodonFreq = 2).

### RrnL and rrnS structures

Secondary RNA structures for the small (*rrnS*) and large (*rrnL*) ribosomal units where modelled based on the proposed *rrnS* structure of the crustacean *Artemia franciscana* (accession number X69067, structure at http://www.rna.ccbb.utexas.edu/RNA/Structures/b.16.m.A.franciscana.bpseq) and the *rnnL* structure of *A. salina* (X12965, http://www.rna.ccbb.utexas.edu/RNA/Structures/d.23.m.A.salina.bpseq; [42]. Ribosomal sequences from metacrangonyctid and *Artemia* species were aligned using MAFFT v6.8 [55] taking into account secondary structure (*xinsi* command). The aligned sequences were folded using as reference the secondary structures of the respective *Artemia* species using RNAsalsa v0.8.1 with default parameters [56]. Folded structures were visualized and refined using the graphic tool VARNA v3.9 (Visualization Applet for RNA, http://varna.lri.fr/index.php?lang=en&css=varna&page=downloads) [57]. Secondary structures were first obtained for major domains separately since global folding approaches artificially joined different domains. MFOLD (http://mfold.rna.albany.edu/?q=mfold/rna-folding-form) [58] was subsequently used to correct secondary structure discrepancies at sequence-conserved regions. In some cases, secondary structures showing suboptimal minimum free energy values were chosen, as they were more similar to those accepted for *Artemia*. Conservation profile of DNA sequences was implemented using the online program PRALINE (http://www.ibi.vu.nl/programs/pralinewww/) [59] using the standard progressive strategy.

### Availability of supporting data

The data set supporting the results of this article is included within the article in Table 1.

## Electronic supplementary material

Additional file 1: **This figure shows nucleotide composition, AT and GC skew values of protein-coding genes for the available crustacean mitogenomes (data from the Metazoan Mitochondrial Genomes Accesible database** http://amiga.cbmeg.unicamp.br **).** Feijao, P.C; Neiva, L.S; Azeredo-Espin, A.M.L. & Lessinger, A.C. (2006). AMiGA: The Arthropodan Mitochondrial Genomes Accessible database. Bioinformatics, 22(7):902–903. Note that all isopods, several crustaceans and all metacrangonyctids have positive GC skews (red line). (PNG 39 KB)

Additional file 2: **This figure shows a positive correlation between the effective number of codons (ENC) of metacrangonyctid mitochondrial protein-coding genes and GC content at third codon positions.** (JPEG 234 KB)

Additional file 3: Table S1: – List of start and stop codons for the mitochondrial protein-coding genes of Metacrangonyctidae. **Table S2.** – List of sequence spacers found in metagrangonyctid mitogenomes with details on their size and location in the corresponding mitochondrial genome. **Table S3.** – Dot-Bracket notation of small and large ribosomal RNA structures of “Metacrangonyx boveii” HE860498. **Table S4.** – Details about species names, codes, sampling localities and accession numbers for the Metagranconyctidae mitogenomes. **Table S5.** – Primers used for long PCR amplifications of Metacrangonyctidae mitogenomes. (XLS 46 KB)

Additional file 4: **Details of the putative secondary structures and sequence motifs found in the mitochondrial control regions of different metacrangonytid species.** See main text for further details. (JPEG 666 KB)

## References

[1] Waeschenbach A, Telford MJ, Porter JS, Littlewood DT (2006). The complete mitochondrial genome of *Flustrellidra hispida* and the phylogenetic position of Bryozoa among the Metazoa. Mol Phylogenet Evol.

[2] Fernández-Silva P, Enriquez JA, Montoya J (2003). Replication and transcription of mammalian mitochondrial DNA. Exp Physiol.

[3] Zhang D-X, Hewitt GM (1997). Insect mitochondrial control region: a review of its structure, evolution and usefulness in evolutionary studies. Biochem Syst Ecol.

[4] Saito S, Tamura K, Aotsuka T (2005). Replication origin of mitochondrial DNA in insects. Genetics.

[5] Bernt M, Bleidorn C, Braband A, Dambach J, Donath A, Fritzsch G, Golombek A, Hadrys H, Jühling F, Meusemann K, Middendorf M, Misof B, Perseke M, Podsiadlowski L, von Reumont B, Schierwater B, Schlegel M, Schrödl M, Simon S, Stadler PF, Stöger I, Struck TH (2013). A comprehensive analysis of bilaterian mitochondrial genomes and phylogeny. Mol Phylogenet Evol.

[6] Schierwater B, Stadler P, DeSalle R, Podsiadlowski L (2013). Mitogenomics and metazoan evolution. Mol Phylogenet Evol.

[7] Zhang P, Zhou H, Chen YQ, Liu YF, Qu LH (2005). Mitogenomic perspectives on the origin and phylogeny of living amphibians. Syst Biol.

[8] Pacheco MA, Battistuzzi FU, Lentino M, Aguilar R, Kumar S, Escalante AA (2011). Evolution of modern birds revealed by mitogenomics: timing the radiation and origin of major orders. Mol Biol Evol.

[9] Morin PA, Archer FI, Foote AD, Vilstrup J, Allen EE, Wade P, Durban J, Parsons K, Pitman R, Li L, Bouffard P, Abel Nielsen SC, Rasmussen M, Willerslev E, Gilbert MT, Harkins T (2010). Complete mitochondrial genome phylogeographic analysis of killer whales (*Orcinus orca*) indicates multiple species. Genome Res.

[10] Wielstra B, Arntzen JW (2011). Unraveling the rapid radiation of crested newts (*Triturus cristatus* superspecies) using complete mitogenomic sequences. BMC Evol Biol.

[11] Krebes L, Bastrop R (2012). The mitogenome of *gammarus duebeni* (crustacea amphipoda): a new gene order and non-neutral sequence evolution of tandem repeats in the control region. Comp Biochem Physiol Part D Genomics Proteomics.

[12] Bauzà-Ribot MM, Juan C, Nardi F, Oromí P, Pons J, Jaume D (2012). Mitogenomic phylogenetic analysis supports continental-scale vicariance in subterranean thalassoid crustaceans. Curr Biol.

[13] Stock JH (1977). The taxonomy and zoogeography of hadziid amphipoda, with emphasis on the west Indian taxa. Stud Fauna Curaçao Caribbean Isl.

[14] Ito A, Aoki MN, Yokobori S, Wada H (2010). The complete mitochondrial genome of *caprella scaura* (crustacea, amphipoda, caprellidea), with emphasis on the unique gene order pattern and duplicated control region. Mitochondrial DNA.

[15] Kilpert F, Held C, Podsiadlowski L (2012). Multiple rearrangements in mitochondrial genomes of isopoda and phylogenetic implications. Mol Phylogenet Evol.

[16] Bauzà-Ribot MM, Jaume D, Juan C, Pons J (2009). The complete mitochondrial genome of the subterranean crustacean *metacrangonyx longipes* (amphipoda): a unique gene order and extremely short control region. Mitochondrial DNA.

[17] Boore JL, Collins TM, Stanton D, Daehler LL, Brown WM (1995). Deducing the pattern of arthropod phylogeny from mitochondrial DNA rearrangements. Nature.

[18] Boore JL, Lavrov DV, Brown WM (1998). Gene translocation links insects and crustaceans. Nature.

[19] Cook CE, Yue Q, Akam M (2005). Mitochondrial genomes suggest that hexapods and crustaceans are mutually paraphyletic. P Roy Soc B Biol Sci.

[20] Shin SC, Cho J, Lee JK, Ahn do H, Lee H, Park H (2012). Complete mitochondrial genome of the Antarctic amphipod *gondogeneia Antarctica* (crustacea, amphipod). Mitochondrial DNA.

[21] Ki JS, Hop H, Kim SJ, Kim IC, Park HG, Lee JS (2010). Complete mitochondrial genome sequence of the arctic gammarid, *onisimus nanseni* (crustacea; amphipoda): novel gene structures and unusual control region features. Comp Biochem Physiol Part D Genomics Proteomics.

[22] Bernt M, Merkle D, Ramsch K, Fritzsch G, Perseke M, Bernhard D, Schlegel M, Stadler P, Middendorf M (2007). CREx: inferring genomic rearrangements based on common intervals. Bioinformatics.

[23] Yang JS, Nagasawa H, Fujiwara Y, Tsuchida S, Yang WJ (2008). The complete mitochondrial genome sequence of the hydrothermal vent galatheid crab *shinkaia crosnieri* (crustacea: decapoda: anomura): a novel arrangement and incomplete tRNA suite. BMC Genom.

[24] Hassanin A, Leger N, Deutsch J (2005). Evidence for multiple reversals of asymmetric mutational constraints during the evolution of the mitochondrial genome of metazoa, and consequences for phylogenetic inferences. Syst Biol.

[25] Kilpert F, Podsiadlowski L (2006). The complete mitochondrial genome of the common sea slater, *ligia oceanica* (crustacea, isopoda) bears a novel gene order and unusual control region features. BMC Genom.

[26] Kilpert F, Podsiadlowski L (2010). The Australian fresh water isopod (phreatoicidea: isopoda) allows insights into the early mitogenomic evolution of isopods. Comp Biochem Physiol Part D Genomics Proteomics.

[27] Wright F (1990). The ‘effective number of codons’ used in a gene. Gene.

[28] Wang Y, Huang XL, Qiao GX (2013). Comparative analysis of mitochondrial genomes of five aphid species (hemiptera: aphididae) and phylogenetic implications. PLoS One.

[29] Giráldez F, Townsend JP (2011). PhyDesign: an online application for profiling phylogenetic informativeness. BMC Evol Biol.

[30] Havird JC, Santos SR (2014). Performance of single and concatenated sets of mitochondrial genes at inferring metazoan relationships relative to full mitogenome data. PLoS One.

[31] Oliveira DC, Raychoudhury R, Lavrov DV, Werren JH (2008). Rapidly evolving mitochondrial genome and directional selection in mitochondrial genes in the parasitic wasp *nasonia* (hymenoptera: pteromalidae). Mol Biol Evol.

[32] Lloyd RE, Foster PG, Guille M, Littlewood DT (2012). Next generation sequencing and comparative analyses of *xenopus* mitogenomes. BMC Genom.

[33] Castellana S, Vicario S, Saccone C (2011). Evolutionary patterns of the mitochondrial genome in metazoa: exploring the role of mutation and selection in mitochondrial protein coding genes. Genome Biol Evol.

[34] Ojala D, Montoya J, Attardi G (1981). tRNA punctuation model of RNA processing in human mitochondria. Nature.

[35] Jühling F, Püz J, Bernt M, Donath A, Middendorf M, Florentz C, Stadler PF (2012). Improved systematic tRNA gene annotation allows new insights into the evolution of mitochondrial tRNA structures and into the mechanisms of mitochondrial genome rearrangements. Nucleic Acids Res.

[36] Cheng N, Mao Y, Shi Y, Tao S (2012). Coevolution in RNA molecules driven by selective constraints: evidence from 5S rRNA. PLoS One.

[37] Chao JA, Patskovsky Y, Almo SC, Singer RH (2008). Structural basis for the coevolution of a viral RNA–protein complex. Nat Struct Mol Biol.

[38] Lavrov DV, Brown WM, Boore JL (2000). A novel type of RNA editing occurs in the mitochondrial tRNAs of the centipede *lithobius forficatus*. Proc Natl Acad Sci U S A.

[39] Bernt M, Braband A, Schierwater B, Stadler PF (2013). Genetic aspects of mitochondrial genome evolution. Mol Phylogenet Evol.

[40] Plazzi F, Ribani A, Passamonti M (2013). The complete mitochondrial genome of *solemya velum* (mollusca: bivalvia) and its relationships with conchifera. BMC Genom.

[41] Doublet V, Helleu Q, Raimond R, Souty-Grosset C, Marcadé I (2013). Inverted repeats and genome architecture conversions of terrestrial isopods mitochondrial DNA. J Mol Evol.

[42] Cannone JJ, Subramanian S, Schnare MN, Collett JR, D’Souza LM, Du Y, Feng B, Lin N, Madabusi LV, Müller KM, Pande N, Shang Z, Yu N, Gutell RR (2002). The comparative RNA web (CRW) site: an online database of comparative sequence and structure information for ribosomal, intron, and other RNAs. BMC Bioinformatics.

[43] Negrisolo E, Babbucci M, Patarnello T (2011). The mitochondrial genome of the ascalaphid owlfly *libelloides macaronius* and comparative evolutionary mitochondriomics of neuropterid insects. BMC Genom.

[44] Carapelli A, Soto-Adames FN, Simon C, Frati F, Nardi F, Dallai R (2004). Secondary structure, high variability and conserved motifs for domain III of 12S rRNA in the *arthropleona* (hexapoda; collembola). Insect Mol Biol.

[45] Carapelli A, Comandi S, Convey P, Nardi F, Frati F (2008). The complete mitochondrial genome of the Antarctic springtail *cryptopygus antarcticus* (hexapoda: collembola). BMC Genom.

[46] Meyer M, Stenzel U, Myles S, Prufer K, Hofreiter M (2007). Targeted high-throughput sequencing of tagged nucleic acid samples. Nucleic Acids Res.

[47] Timmermans M, Dodsworth S, Culverwell L, Bocak L, Ahrens D, Littlewood DT, Pons J, Vogler AP (2010). Why barcode? High-throughput multiplex sequencing of mitochondrial genomes for molecular systematics. Nucleic Acids Res.

[48] Bernt M, Donath A, Jühling F, Externbrink F, Florentz C, Fritzsch G, Pütz J, Middendorf M, Stadler PF (2013). MITOS: improved de novo metazoan mitochondrial genome annotation. Mol Phylogenet Evol.

[49] Tamura K, Peterson D, Peterson N, Stecher G, Nei M, Kumar S (2011). MEGA5: molecular evolutionary genetics analysis using maximum likelihood, evolutionary distance, and maximum parsimony methods. Mol Biol Evol.

[50] Perna NT, Kocher TD (1995). Patterns of nucleotide composition at fourfold degenerate sites of animal mitochondrial genomes. J Mol Evol.

[51] Supek F, Vlahovicek K, INCA (2004). Synonymous codon usage analysis and clustering by means of self-organizing map. Bioinformatics.

[52] Rice P, Longden I, Bleasby A (2000). EMBOSS: the European molecular biology open software suite. Trends Genet.

[53] Goldman N, Yang Z (1994). A codon-based model of nucleotide substitution for protein-coding DNA sequences. Mol Biol Evol.

[54] Yang Z (2007). PAML 4: a program package for phylogenetic analysis by maximum likelihood. Mol Biol Evol.

[55] Katoh K, Toh H (2008). Recent developments in the MAFFT multiple sequence alignment program. Brief Bioinform.

[56] Stocsits RR, Letsch H, Hertel J, Misof B, Stadler PF (2009). Accurate and efficient reconstruction of deep phylogenies from structured RNAs. Nucleic Acids Res.

[57] Darty K, Denise A, Ponty Y (2009). VARNA: interactive drawing and editing of the RNA secondary structure. Bioinformatics.

[58] Zuker M (2003). Mfold web server for nucleic acid folding and hybridization prediction. Nucleic Acids Res.

[59] Simossis VA, Heringa J (2005). PRALINE: a multiple sequence alignment toolbox that integrates homology-extended and secondary structure information. Nucleic Acids Res.

