# Supplementary figures and images for: Next-generation sequencing, phylogenetic signal and comparative mitogenomic analyses in Metacrangonyctidae (Amphipoda: Crustacea)
Source: BMC Genomics. 2014 Jul 6;15(1):566. doi: 10.1186/1471-2164-15-566 (PMC4112215; doi:10.1186/1471-2164-15-566)

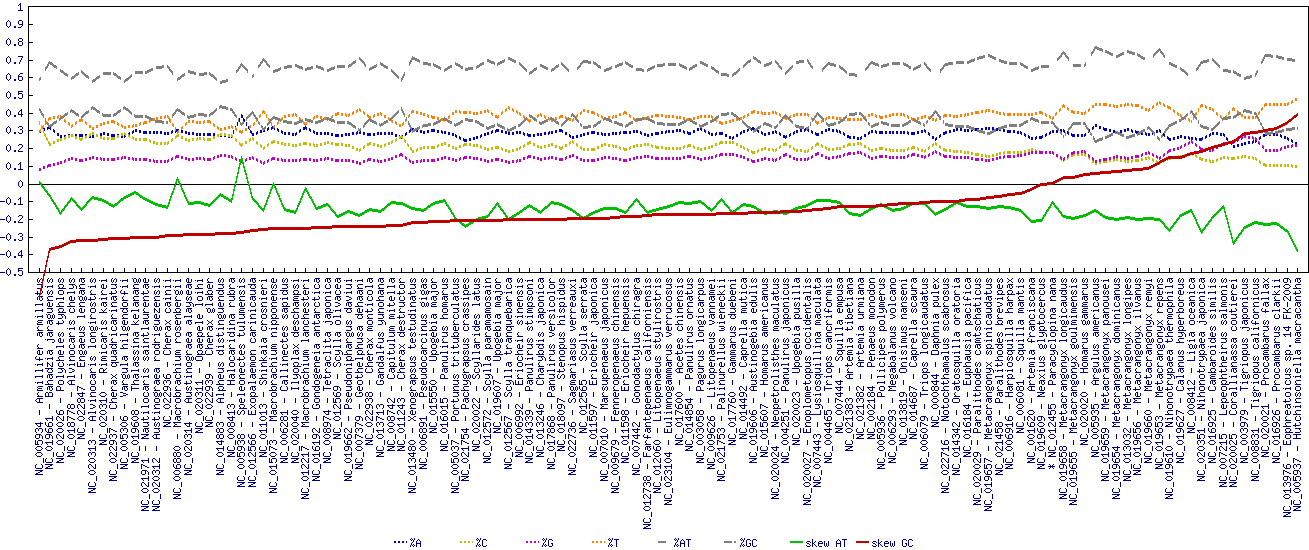

Supplement: Supplementary file 1 — Additional file 1: This figure shows nucleotide composition, AT and GC skew values of protein-coding genes for the available crustacean mitogenomes (data from the Metazoan Mitochondrial Genomes Accesible database http://amiga.cbmeg.unicamp.br ). Feijao, P.C; Neiva, L.S; Azeredo-Espin, A.M.L. & Lessinger, A.C. (2006). AMiGA: The Arthropodan Mitochondrial Genomes Accessible database. Bioinformatics, 22(7):902–903. Note that all isopods, several crustaceans and all metacrangonyctids have positive GC skews (red line). (PNG 39 KB) [file 12864_2014_6283_MOESM1_ESM.png]

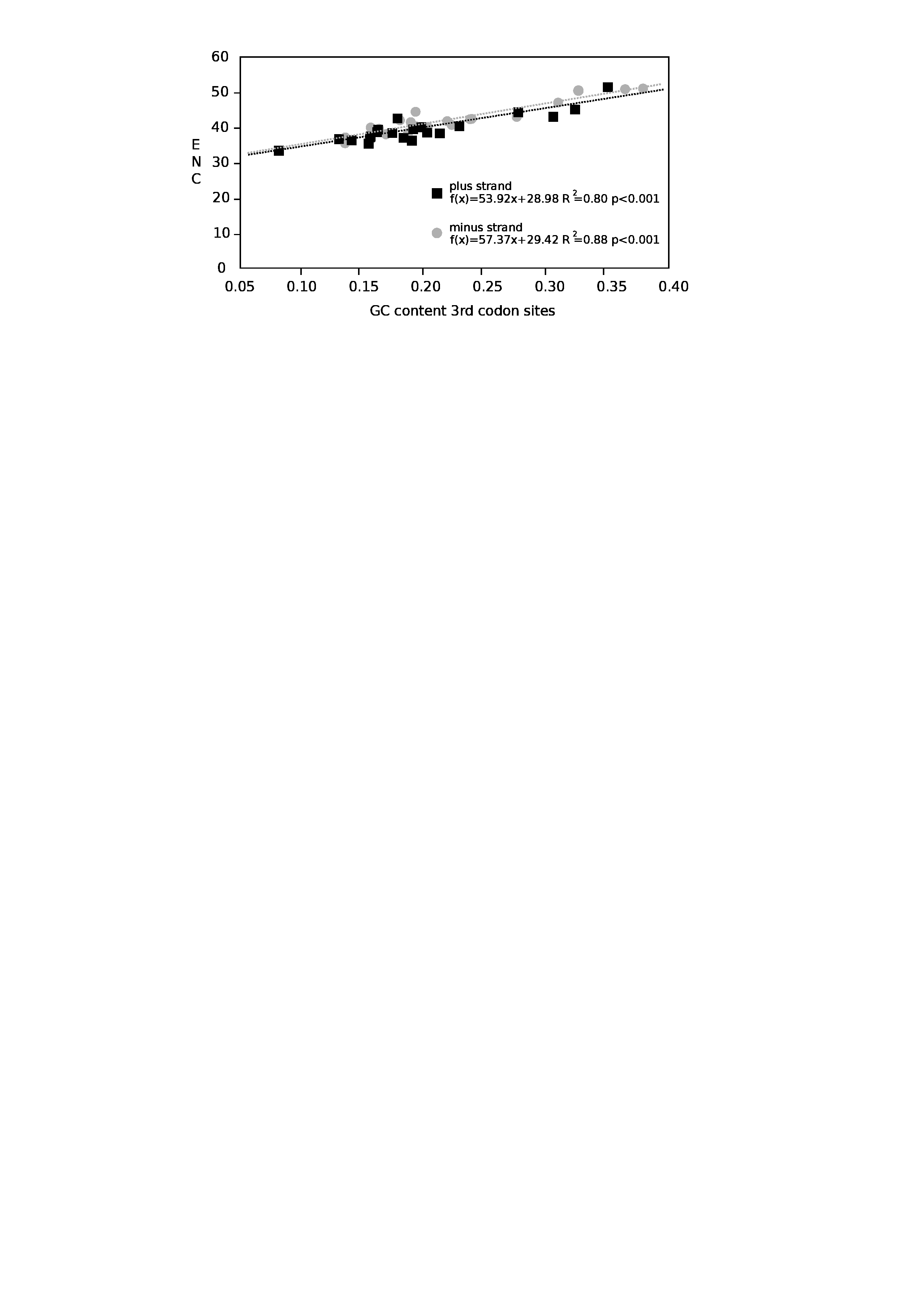

Supplement: Supplementary file 2 — Additional file 2: This figure shows a positive correlation between the effective number of codons (ENC) of metacrangonyctid mitochondrial protein-coding genes and GC content at third codon positions. (JPEG 234 KB) [file 12864_2014_6283_MOESM2_ESM.jpeg]

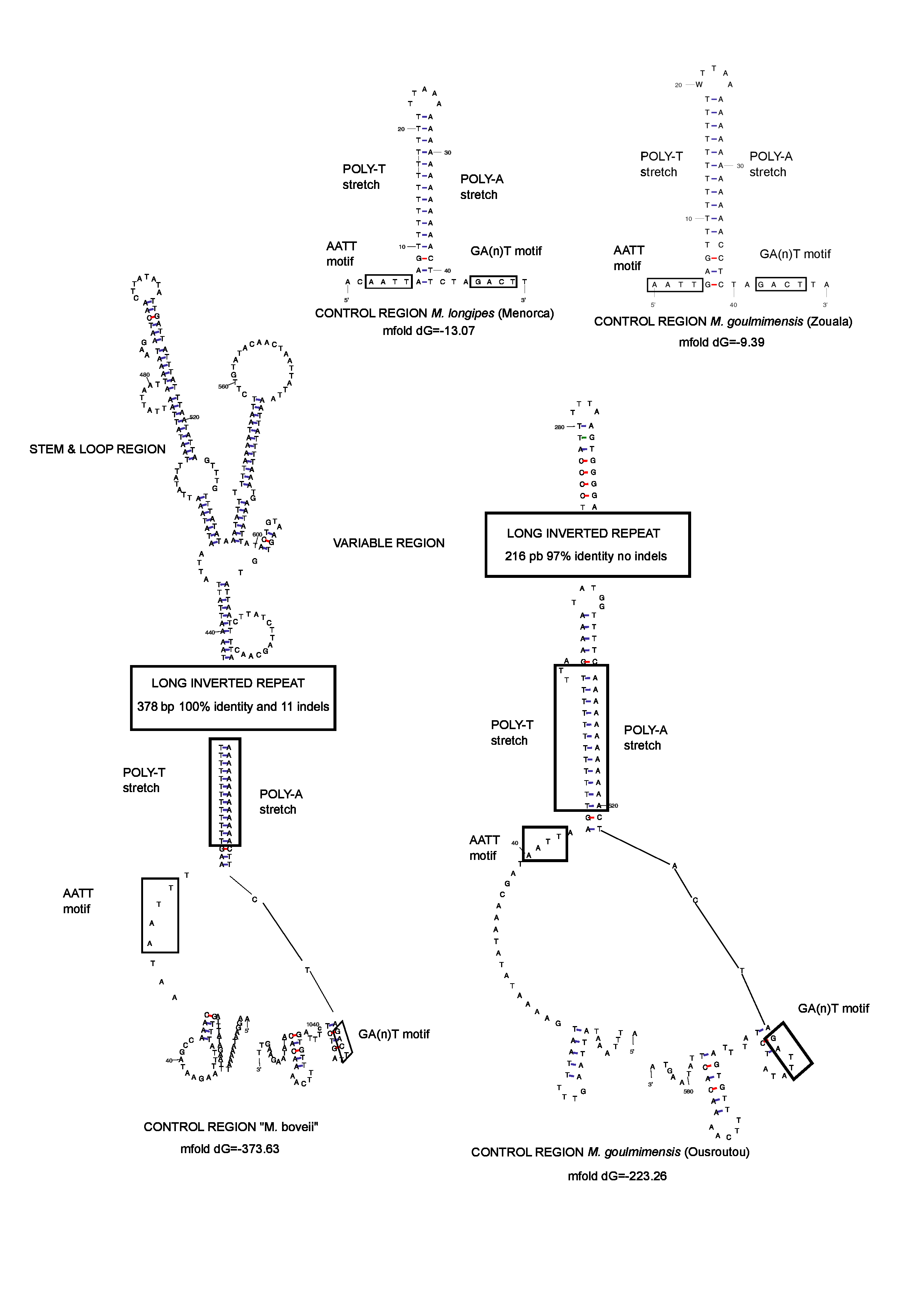

Supplement: Supplementary file 4 — Additional file 4: Details of the putative secondary structures and sequence motifs found in the mitochondrial control regions of different metacrangonytid species. See main text for further details. (JPEG 666 KB) [file 12864_2014_6283_MOESM4_ESM.jpeg]
